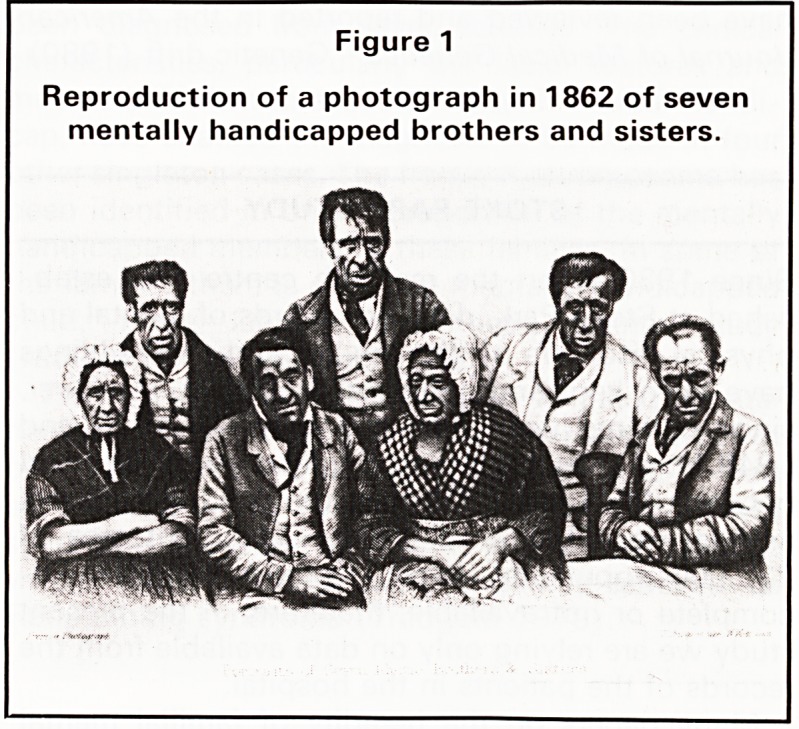# Familial Mental Handicap
*Abbreviated version of the paper presented at the 6th International Congress of the International Association for the Scientific Study of Mental Deficiency, Toronto, Canada (August 1982).


**Published:** 1983

**Authors:** J. Jancar

**Affiliations:** Consultant Psychiatrist, Stoke Park Hospital, Bristol, and Clinical Lecturer in Mental Health (Mental Handicap), University of Bristol


### Familial Mental Handicap*

**J. Jancar, M.B., B.Ch, B.A.O., F.R.C.Psych., D.P.M.**
Consultant Psychiatrist, Stoke Park Hospital, Bristol, and Clinical Lecturer in Mental Health (Mental Handicap), University of Bristol

### INTRODUCTION

One hundred and twenty years ago, Dr. Browne, Commissioner of Lunacy for Scotland, sent the following annotation "Group of seven Idiots, brother and sisters, from a Photograph" to the *Journal of Mental Science* (1862):

> 'In passing through an asylum I saw five odd and apparently aged men, seated together around a table and apart from the other patients. They smiled; spoke a few words; gabbled or jargonised. My companion said, "They like to dine together". On complimenting him for his attention to their wishes, he answered, "Oh, they are all brothers". On going to the department for females, I observed two quiet, elderly women, indulged in the same way. "These", said my guide, "are sisters, and sisters of the five brothers. They were the children of poor but industrious and self-supporting parents, who were somewhat eccentric, and believed to be cousins, or related. They are all, in different degrees, imbecile, ineducable, irresponsible, and incapable of guiding or maintaining themsevles. They had, besides, a brother who disappeared, and who was supposed to have been drowned in a quarry; another imbecile sister still alive; and two brothers and one sister, who were healthy."' (Figure 1.)

Since this annotation many papers and surveys on the subject of familial mental handicap have been published from various centres. The results of the studies varied from place to place depending on the sample of population studied and the investigator's approaches.

Shuttleworth and Beach in 1892 recorded statistics from 1200 cases observed at the Royal Albert Asylum and 1180 cases at Darenth Asylum, and stated that 21.38% of patients had hereditary mental weakness (insanity or imbecility).

In the USA Barr (1904) compiled 3050 cases of mental deficiency from various sources, but chiefly from the records of the Pennsylvania Training School, and found a family history of Idiocy and Imbecility in 27.38% of patients.

Following the result of an enquiry into a large number of cases of all ages, degrees and types, Tredgold (1952) came to the conclusion that approximately 80% were suffering from 'amentia' due to inheritance and 20% from 'amentia' due to the environment.

**Figure 1**

**Reproduction of a photograph in 1862 of seven mentally handicapped brothers and sisters.**

He also produced a table of known reports on the heredity of mental handicap, including Penrose Colchester's Survey, which revealed a wide range of results from 29% to 90%.

The most important survey in the field was 'A Clinical and Genetic Study of 1,280 Cases of Mental Defect' by Penrose in 1938. He, among other things, reported an excess of males among mentally handicapped patients.

Over the years families with clearly X-linked pedigrees have been reported by, for example, Martin and Bell (1943) and Renpenning et al (1962). Davison (1973) first suggested that X-linked genes should be considered in the aetiology of non-specific mental handicap as a whole, but it was Turner and Turner (1974) who stressed the importance of this. They further concluded that X-linked genes were responsible for the condition in 20% of affected males in New South Wales. More recent studies by Herbst

*Abbreviated version of the paper presented at the 6th International Congress of the International Association for the Scientific Study of Mental Deficiency, Toronto, Canada (August 1982).

and Miller (1980) in British Columbia gave an incidence of at least 1.83 per 1000 males for X-linked mental handicap.

The new chapter on X-linked mental handicap began in 1969 when Lubs reported the presence of a marker X-chromosome in affected males belonging to a family whose pedigree indicated X-linked mental handicap.

There was no further progress made until 1977 when Harvey et al reported an identical marker X-chromosome in a portion of metaphases from males of four such families. Sutherland (1977) showed that this marker was in fact a 'fragile site' occurring at band q 27 or q 28 of the X-chromosomes. He pointed out that the culture conditions were critical if the fragile site on the X-chromosome were to be expressed.

The studies mentioned so far, and many others, have been reviewed and reported in the *American Journal of Medical Genetics* – Genetic drift (1980).

### STOKE PARK STUDY

Since 1930 when the research centre was established at Stoke Park, detailed records of mental and physical disorders of the families and their siblings have been kept and constantly updated. However, since patients were admitted between 1909 and 1948 from every county of England and Wales and between 1948 and 1974 from the West of England, and at present, from a district catchment area of 222,000 population, family histories are often incomplete or not available; therefore, in the present study we are relying only on data available from the records of the patients in the hospital.

Many papers on the heredity of familial mental handicap, X-linked conditions, biochemical and chromosomal abnormalities and other disorders were published at Stoke Park (Jancar, 1981).

*The present study* is an analysis of randomly collected, 1000 families, whose children were admitted to the Stoke Park Group of Hospitals, with special emphasis on the families with more than one mentally handicapped child.

It was found that members of families were affected as shown in Table 1.

The Lyon hypothesis (1962) in part explains the preponderance of males affected by mental handicap (Table 2).

It is interesting to note that by far the commonest combination of affected siblings was in families with one male and one female mentally handicapped member (62%), whereas in total males were only slightly more numerous than females.

*Congenital Syphilis,* in the pre-antibiotic era, affected 12 families with more than one mentally

**Table 1**

| | |
|---|---|
| Families with only one member affected | 591 = 59.1% (Females 209 = 20.9%) (Males 382 = 38.2%) |
| Families with more than one male affected | 76 = 7.6% |
| Families with more than one female affected | 54 = 5.4% |
| Families with both sexes affected | 279 = 27.9% |
| | 1000 100.0% |

| *Families with males only affected* | | |
|---|---|---|
| 2 males | 68 families | 136 offspring |
| 3 males | 7 families | 21 offspring |
| 4 males | — | — |
| 5 males | 1 family | 5 offspring |
| Total | 76 families | 162 offspring |

| *Families with females only affected* | | |
|---|---|---|
| 2 females | 46 families | 92 offspring |
| 3 females | 7 families | 21 offspring |
| 4 females | 1 family | 4 offspring |
| 5 females | — | — |
| Total | 54 families | 117 offspring |

**Table 2**

**Families with affected siblings of both sexes**

| *Males* | / *Females* | *Number of Families* | *(%)* | *Total Siblings* |
|---|---|---|---|---|
| 1 | 1 | 173 | 62.0 | 346 |
| 2 | 1 | 49 | 17.6 | 147 |
| 1 | 2 | 27 | 9.7 | 81 |
| 1 | 3 | 10 | 3.6 | 40 |
| 3 | 1 | 9 | 3.3 | 36 |
| 4 | 1 | 5 | 1.8 | 25 |
| 4 | 2 | 2 | 0.7 | 12 |
| 2 | 2 | 2 | 0.7 | 8 |
| 2 | 3 | 1 | 0.3 | 5 |
| 1 | 4 | 1 | 0.3 | 5 |
| Total | | 279 | 100.0% | 705* |

* Males, 370; females, 375.

handicapped sibling:

| | |
|---|---|
| Males only | 1 family |
| Females only | 4 families |
| Males and females | 7 families |
| Total | 12 families |

*Consanguinity* (First Cousins) was traced in:

| | |
|---|---|
| Males only | 3 families |
| Females only | 1 family |
| Males and females | 4 families |
| Total | 8 families |

**DOWN'S SYNDROME**

There were 197 cases of Down's syndrome amongst the 1000 families – 132 males and 65 females. Family history of Down's syndrome was noted in three families with males only affected.

In the *families with more than one male,* two examples of X-linked conditions are presented:

(1) *Norrie's disease* (Recessive, sex-linked, progressive oculocerebral degeneration)
Two brothers with Norrie's disease, who were severely mentally handicapped, epileptic and died at Stoke Park Hospital from pulmonary tuberculosis, had 12 male relatives in five generations, who had all suffered from Norrie's disease (Jancar 1980).

(2) *Lesch-Nyhan syndrome* (Familial Hyperuricaemia)
The propositus of the first Bristol family discovered to suffer from this disease died last July at the age of 33. He had two brothers and a nephew afflicted with the same disorder characterised by severe mental handicap, self mutilation, choreoathetotic movements and history of epilepsy (Jancar and Wiley 1973).

An example of the *families with more than one handicapped female* is familial tapetoretinal degeneration. There are three mentally handicapped sisters and a great niece similarly affected who has in addition cerebellar ataxia, epilepsy and deafness. There are other disorders in this family. The mother and one sister suffered from rheumatoid arthritis; the father and his grandson suffered from diabetes mellitus; the youngest sibling had a patent intraventricular septum; and two nephews are mentally handicapped. One miscarriage and one infant death are noted in the same generation (Jancar and Walters 1974).

Two examples of the *families with male and female siblings* are as follows:

(1) Female, severely mentally handicapped with history of superimposed psychotic episodes, retinitis pigmentosa, deafness and generalised dyskinesia, had an older brother who suffered from blindness and a degree of deafness. The patient's younger brother was deaf and dumb and partially sighted (Jancar 1970).

(2) Brother and sister suffering from phenylketonuria, severe mental handicap and epilepsy. Both patients have, in addition, bilateral calcified choroid plexus, and their paternal grandfather suffered from epilepsy.

**FRAGILE X-LINKED MENTAL HANDICAP**

Since 1980 when the new techniques for demonstrating the presence of the fragile X marker chromosome became available we have been re-examining cases with X-linked familial mental handicap. So far 18 cases, including one female, have been diagnosed from seven families. The clinical characteristics, particularly the facial features and macro-orchidism in association with mental handicap, have enabled the diagnosis to be made in four other singleton cases. The fragile X chromosome has been identified in only a proportion of the mentally handicapped members of these families. In some of the families there is only one mentally handicapped child; these families also need further study (McDermott and Walters 1982).

Two families in which fragile X-linked mental handicap was confirmed had to be reclassified. In one, the siblings – three males and one female – had all the signs and symptoms of Rubinstein–Taybi syndrome (Jancar 1965), and in the other family, where three brothers were originally labelled as Renpenning syndrome (Tredgold 1979).

**EXAMPLES OF RARE SYNDROME**

The following rare syndromes were noted in the survey of 1000 families:

Mandibulo-facial dysostosis – (two brothers),
Prader–Willi syndrome – (two brothers),
Sturge–Weber syndrome – (male and two mentally handicapped, epileptic cousins),
Marfan syndrome – (mother and daughter),
Hurler syndrome – (two brothers and one cousin), and
Right-sided hemiplegia – (two brothers).

**PSYCHIATRIC ASPECTS**

It was frequently noted among 1000 families, particularly in the families with Down's syndrome siblings, that there was an occurrence of a variety of mental disorders. These mental disorders are, at present, the subject of a separate study. The importance of a study of psychiatric disorder in relatives of mentally handicapped patients must be emphasised. Penrose in 1966 stated: 'The various branches of psychiatry all have potential value for each other. Provided that we do not neglect or despise the data derived from one another's discipline, there are advances to be made in the diagnosis, prevention and treatment of apparently most intractable mental disease.'

### CONCLUSION

The studies and surveys, including the Stoke Park Study, of familial mental handicap can be grouped into four areas:

(1) *Pre-Penrose Survey (1938)*
When pioneers in the field of mental handicap accumulated data proving that heredity plays a major role in causation of mental handicap.

(2) *Post-Penrose Survey*
Penrose reported in his survey an excess of males among mentally handicapped patients. This observation stimulated a number of workers to study X-linked pedigrees and they came to the conclusion that X-linked genes were responsible for non-specific mental handicap in males.

(3) *Post-Lubs Era*
Since Lubs reported in 1969 the presence of a marker X-chromosome in affected males, in X-linked mental handicap pedigree, new exciting developments have taken place particularly with the discovery of Fragile X chromosome by Sutherland.

The Fragile X has been found in autistic children and by amniotic tap for prenatal diagnosis. Fragile sites in other chromosomes have been described which may prove to be important in the future studies of mental handicap.

(4) *Era of Treatment*
Lejeune (1982) in a letter to the *Lancet* stated that fragility of the X-chromosome can be rectified in vitro by thymine, folic acid, 5-formyltetrahydrofolate, or even amino-acids, precursors of 'monocarbons' and he postulated that monocarbon disorder could be a major cause of mental deficiency, and he suggested an interesting possibility of treating the pregnant Fragile X women in view of his experience of treating a Fragile X $1\frac{1}{2}$ year old child with folic acid.

Lejeune concluded his letter:

> 'Research on the treatment of Fragile X patients has only just begun but this could be the first example in history of cytogenetics where a chromosome associated disease related to a partly-understood chemical abnormality has proved amenable to treatment. Preliminary findings give rise to the hope that cytogenetics will some day become another chapter of true medicine – that is, curative medicine.'

The message for the future is quite clear. It indicates that when no causes are known, cases should be examined and re-investigated. Cytogenetic and other findings may provide a base for genetic counselling, and known or still to be discovered treatment.

### SUMMARY

One thousand families, randomly collected, whose mentally handicapped children were admitted to Stoke Park Group of Hospitals, were studied. The findings, relating to the number of affected siblings, sex predominance, congenital syphilis, consanguinity, fragile X-linked mental handicap, Down's syndrome and other rare syndromes, were analysed and where appropriate illustrated with a few examples. The psychiatric aspect of familial mental handicap has been emphasised. The relevant literature on the subject of familial mental handicap was reviewed.

### ACKNOWLEDGEMENTS

I wish to thank Miss D. Sperin-West, Research Assistant and Psychologist, who has worked for over 40 years at Stoke Park, for invaluable documentation, psychological examinations of the patients and help with researching relevant data; Dr. Graham Carter of Stoke Park Hospital for the help with statistical analysis; Dr. Alan McDermott, Regional Cytogeneticist, Southmead Hospital, Bristol for cytogenetic examination; and Miss Deborah K. Britt for the secretarial work.

### REFERENCES

BARR, W. (1904) Mental defectives–their history, treatment and training. London, Rebman Limited.

BROWNE, F. W. A. (1982) Group of seven idiots, brothers and sisters, from a photograph. *J.Ment.Sci.* **viii**, 429–430.

DAVISON, C. B. C. (1973). Genetic studies in mental subnormality. 1. Familial idiopathic severe subnormality: the question of a contribution by X-linked genes. *Brit.J.Psychiat.* Special Publication No. 8.

GENETIC DRIFT – *American Journal of Medical Genetics* (1980) **7**, 405–505.

HARVEY, J., JUDGE, C. and WIENER, S. (1977) Familial X-linked mental retardation with an X-chromosome abnormality *J.Med.Genet.* **14**, 46–50.
HERBST, D. S. and MILLER, J. R. (1980) Nonspecific X-linked mental retardation II: the frequency in British Columbia. *J.Med.Genet.* **7**, 461–469.
JANCAR, J. (1965) Rubinstein–Taybi's syndrome. *J.Ment. Defic.Res.* **9**, 265–270.
JANCAR, J. (1970) Norrie's disease. Recessive, sex-linked, progressive, oculo-cerebral degeneration. Clinical Genetics, **1**, 353–356.
JANCAR, J. (1970) Retinitis pigmentosa with mental retardation, deafness and XX/XO chromosomes. *J.Ment. Defic.Res.* **14**, 269–273.
JANCAR, J. (1981) Research at Stoke Park Mental Hospital (1930–1980). The Supplement to Stoke Park Studies. Dorchester, Henry Ling Ltd., Dorset Press.
JANCAR, J. and Walters, R. M. (1974) Tapeto-retinal degeneration in three mentally retarded sisters with other disorders in the family tree. *Acta.Genet.Med. Gemellol.*(Roma) **23**, 175–180.
JANCAR, J. and WILEY, Y. (1973) Lesch–Nyhan syndrome (familial hyperuricaemia). *Acta.Univ.Carol.(Med. Monogr.) (Praha)* **56**, 183–185.
LEJEUNE, J. (1982) Is the fragile X syndrome amenable to treatment? *Lancet* **1**, 273–274.
LUBS, H. A. (1969) A marker-X chromosome. *Am.J.Hum. Genet.* **21**,231–244.
LYON, M. F. (1962) Sex chromatin and gene action in the mammalian X-chromosome. *Am.J.Hum.Genet.* **14**, 135–148.
McDERMOTT, A. and WALTERS, R. M. (1982) Personal communication.
MARTIN, J. P. and BELL, J. (1943) A pedigree of mental defect showing sex-linkage. *J.Neurol.Psychiat.* **6**, 154–157.
PENROSE, L. S. (1938) A clinical and genetic study of 1,280 cases of mental defect (The 'Colchester Survey'). Special Report Series No. 229. Medical Research Council, London.
PENROSE, L. S. (1966) The contribution of mental deficiency research to psychiatry. *Brit.J.Psychiat.* **112**, 747–755.
RENPENNING, H., GERRARD, J. W., ZALESKI, W. A. and TABATA, T. (1962) Familial sex-linked mental retardation. *Canad.Med.Ass.J.* **87**, 954–956.
SUTHERLAND, G. R. (1977) Fragile sites on human chromosomes. Demonstration of their dependence on the type of tissue culture medium. *Science* **197**, 265–266.
SHUTTLEWORTH, G. E. and BEACH, F. (1982) Articles on idiocy – etiology. In Hack Tuke's Dictionary of psychological medicine. London.
TREDGOLD, A. F. (1952) A text-book of mental deficiency (amentia), 8th ed. London, Baillière, Tindall and Cox.
TREDGOLD'S MENTAL RETARDATION (1979) Craft M. (ed.), p. 108, 12th ed. London, Baillière, Tindall.
TURNER, G. and TURNER, B. (1974) X-linked mental retardation. *J.Med.Genet.* **11**, 109–113.

## Figures and Tables

**Figure 1 f1:**